# Nutritional strategies of physically active subjects with muscle dysmorphia

**DOI:** 10.1186/1755-7682-6-25

**Published:** 2013-05-26

**Authors:** Nadir Contesini, Fernando Adami, Márcia de-Toledo Blake, Carlos BM Monteiro, Luiz C Abreu, Vitor E Valenti, Fernando S Almeida, Alexandre P Luciano, Marco A Cardoso, Jucemar Benedet, Francisco de Assis Guedes de Vasconcelos, Claudio Leone, Deivis Elton Schlickmann Frainer

**Affiliations:** 1 Uniasselvi/FAMEBLU, R Dr Pedro Zimmermann, 385 89065-000, Blumenau, SC, Brazil; 2 Laboratory of Scientific Writing, Department of Morphology and Physiology, School of Medicine of ABC, Av. Príncipe de Gales, n 821 09060-650, Santo André, SP, Brazil; 3 School of Arts, Sciences and Humanities, USP, Av. Arlindo Béttio, 1000 Ermelino Matarazzo, 03828-000, São Paulo, SP, Brazil; 4 Post-graduation Program in Physiotherapy, Faculty of Sciences and Technology, UNESP, Rua Roberto Simonsen, 305 19060-900, Presidente Prudente, SP, Brazil; 5 Post-graduation Program in Physical Education, UFSC, Campus Universitário Reitor João David Ferreira Lima, 88040-900, Florianópolis, SC, Brazil; 6 Post-graduation Program in Nutrition, UFSC, Campus Universitário Reitor João David Ferreira Lima, 88040-900, Florianópolis, SC, Brazil

**Keywords:** Diet, Nutrition, Physical exercise, Muscle dysmorphia

## Abstract

**Background:**

The aim of this study was to identify dietary strategies for physically active individuals with muscle dysmorphia based on a systematic literature review.

**Method:**

References were included if the study population consisted of adults over 18 years old who were physically active in fitness centers. We identified reports through an electronic search ofScielo, Lilacs and Medline using the following keywords: muscle dysmorphia, vigorexia, distorted body image, and exercise. We found eight articles in Scielo, 17 in Medline and 12 in Lilacs. Among the total number of 37 articles, only 17 were eligible for inclusion in this review.

**Results:**

The results indicated that the feeding strategies used by physically active individuals with muscle dysmorphia did not include planning or the supervision of a nutritionist. Diet included high protein and low fat foods and the ingestion of dietary and ergogenic supplements to reduce weight.

**Conclusion:**

Physically active subjects with muscle dysmorphia could benefit from the help of nutritional professionals to evaluate energy estimation, guide the diet and its distribution in macronutrient and consider the principle of nutrition to functional recovery of the digestive process, promote liver detoxification, balance and guide to organic adequate intake of supplemental nutrients and other substances.

## Background

Muscle dysmorphia (MD) is defined as an obsessive-compulsive disorder, both for the obsession with musculature, and the compulsion to physical exercise. It produces important changes in feeding behavior, resulting in radical diets and the use of dietary supplements in order to compensate or to obtain muscle mass and to reduce body fat mass [1-3].

This disorder presented by physically actives subjects, often lifters and bodybuilders, was initially described by Pope et al. [4] as anorexia nervosa. The change in the nomenclature to muscle dysmorphia was through the observation that the malfunctions were not undernutrition, but in the image of the muscle composition [5-8].

Although it is not yet recognized as a disease by the manual classification in the ICD-10 and DSM-IV, what is known about the prevalence of MD in Brazil is through estimates from other studies. In 2001, Olivardia, Pope Junior and Hudson observed that regular gym participants who practice muscle-building had a higher prevalence of muscle dysmorphia and anabolic steroid use than individuals who do not practice muscle building. The prevalence of MD among lifters is estimated to be between 10% and 53% worldwide, whereas the prevalence in Chile is estimated to range from 10.1% to 20% [2,6]. The MD affects more men than women, especially adolescents and young male adults, who also have a higher prevalence of anabolic steroid use that may reach 2% [9].

We conducted systematic literature review of scientific studies conducted between 2001 and 2011 to learn about the characteristics of the food and nutritional diet of subjects with MD. This knowledge is important to understand and intervene in eating disorders related to the distorted view of one's body as well as their feeding strategies motivated by the desire for short term aesthetic modification, in search for the perfect body.

## Methods

We conducted a systematic review of the SciELO, MEDLINE and Lilacs databases for the years 2001 to 2011. The keywords used in the search were: muscle dysmorphia, distorted body image, physical exercises, and diet and nutrition (used in comination). Our selection criteria required that the research participants should be physically active, the title or abstract of the manuscript contained the word “muscle dysmorphia” and that the articles would focus their objectives on the study of specific food habits related to MD. Literature review studies were excluded.

Among the 37 articles found, only 17 met the selection criteria These 17 articles made direct reference specifically to the consumption of food by physically active subjects predisposed to MD.

From the selected articles, four are listed in Scielo database [3,5,7,10], four in Lilacs database [1,5,6,9] and the other 9 in Medline database [2,4,8,11-16]. All these publications were in English except two which were in Spanish [2,16].

## Results

The main characteristics of the 17 studies selected in this review are summarized in Tables 1, 2, 3, 4, 5, 6 and 7 identifying the author(s) and year of publication, population and sample, and a summary of the main results. The studies analyzed in this study show that most physically active subjects in gyms follow specific diets aimed to gain muscle mass and lose fat, but this does not mean that diets were properly followed. It was evident in the results of the research, that most physically active individuals prepare their own diets randomly, without specific nutritional knowledge or guidance by professionals [2,3,17].

**Table 1:** Description of quantities of articles analyzed through systematic review

**Total of studies found**	**Type of study**	**N**^**r**^	**%**
Ineligible	Literature review	12	
	Without mentioning diets	8	
Partial		20	54,05
Eligible	Dietary questionnaires with physically actives subjects	11	
	Consumption of ergogenics	6	
Partial	17	45,94	
Total survey	Generalized	37	100%

**Table 2:** Main characteristics of the studies that were included in the systematic review

**Author/Year**	**Population/Sample**	**Results**
Olivardia; Pope Jr.; Hudson (2001)	24 men with MD and 30 without MD	Athletes with MD have more body dissatisfaction, different eating attitudes, consume more anabolic and have more eating disorders than weight lifters without MD
Pope (et al., 2005)	63 men physically active subjects at fitness centers in Chicago, USA	14 men have been confirmed with MD. These are likely to have attempted suicide, poorer quality of life, greater frequency of substance use disorder and anabolic steroid abuse
Kanayama (et al., 2006)	89 men weightlifters from Massachusetts and Florida were evaluated upon symptoms of MD	Consumption of anabolic steroids among men with MD reveals the quest for the perfect body which leads to a stereotyped view of manhood

**Table 3:** Main characteristics of the studies that were included in the systematic review

**Author/Year**	**Population/Sample**	**Results**
Linhares e Lima (2006)	334 bodybuilders from 4 major gyms of Campos dos Goytacazes/RJ	65% consume dietary supplements, proteins and amino acids often without guidance from professionals to increase muscle mass and get quick results. Stress, high blood pressure, euphoria, abdominal pain, drowsiness and low immunity are some of the most common symptoms
Pereira; Souza; Lisbôa, 2007	141 bodybuilders who responded to a questionnaire	Respondents have certain knowledge to identify food sources of macronutrients and that fasting is not the best way to reduce body weight. There was lack of knowledge about the use of supplements due to the orientation for consumption is not specialized
Pereira; Cabral, 2007	26 women bodybuilders over the age of 50 years at a fitness center in Recife (PE)	The majority (61.5%) had normal weight, but all had no adequate power with regard to the variety and quantity of food. The study highlights the risk of nutritional deficiencies in the diet unbalanced in physically actives subjects

**Table 4:** Main characteristics of the studies that were included in the systematic review

**Author/Year**	**Population/Sample**	**Results**
Pinto e Araújo (2007)	15 men between 20 and 35 regulars at a gym academy in the city of Caratinga practicing bodybuilding with the objective of muscle hypertrophy	Physically active subjects cultivate common sense that eating a lot of protein and little fat is healthy, eliminates fat mass and gains lean tissue, they feed on little food and no proper combination of nutrients, compensating with supplements for fast results
Hay (et al., 2008)	3001 respondents in 1995 and 3047 in 2005 in urban and rural areas of Australia	There was a change in eating habits, but the growth of bulimia and anorexia was irrelevant
Sardinha; Oliveira; Araújo, 2008	100 strength training physically actives men between 18 and 35 years	These results confirm a potential applicability of anthropometric indicator valid for the diagnosis of MD, although their application does not replace psychological diagnoses and clinical procedures. These are indicators that help the diagnosis of MD and nutritionists to develop dietary intervention

**Table 5:** Main characteristics of the studies that was included in the systematic review

**Author/Year**	**Population/Sample**	**Results**
Silva Junior (et al., 2008)	120 physically active subjects 14 to 41 years old (77 men and 43 women) in a gym academy in Rio de Janeiro	Vitamins, amino acids and diet pills are the most consumed without prescription. Half (50%) use anabolic steroids for muscle hypertrophy
Iriart; Chaves; Orleans (2009)	Ethnographic qualitative study with 43 male bodybuilders in Salvador (BA) who used anabolic; participative observation	Food supplements and supplements have priority among physically active subjects to the detriment of a balanced diet with carbohydrates, fats and proteins in the ideal amount
Trog e Teixeira (2009)	63 bodybuilders (39 men and 24 women) in 4 gyms of Irati (PR) aged between 19 and 27 years	Most men make use of dietary supplements, and women in a lower percentage. Many recognize the health risks, admit to feel harmed, but continue to increase muscle mass

**Table 6:** Main characteristics of the studies that were included in the systematic review

**Author/Year**	**Population/Sample**	**Results**
Behar e Molinari (2010)	Cross evaluation of 88 male weightlifters of fitness centers in Viña del Mar and Valparaiso (Chile)	Weightlifters spend more time caring for the body than the students, 42% have MD and 67% use anabolic steroids
Damasceno (2010)	Systematic review of 64 studies on the subject	Physically active subjects do not eat a balanced diet and consume great quantities of dietary supplements and anabolic steroids
Trog e Teixeira (2009)	63 bodybuilders (39 men and 24 women) in 4 gyms of Irati (PR) aged between 19 and 27 years	Most men make use of dietary supplements, and women in a lower percentage. Many recognize the health risks, admit to feel harmed, but continue to increase muscle mass

**Table 7:** Main characteristics of the studies that was included in the systematic review

**Author/Year**	**Population/Sample**	**Results**
Sabino; Luz; carvalho, 2010	Bodybuilders of 12 academies of northern and southern Rio de Janeiro	The bodybuilders are switching to a balanced diet supplements in order to gain more muscle mass
Azevedo (et al., 2011)	10 resistance training physically active subjects in gyms in João Pessoa (PB)	The majority is concerned with the body, although it is dissatisfied and modified eating habits, feed improperly, with supplements, exercises and many fewer meals a day in search of the perfect body

Many bodybuilders searched the internet, consulted friends and colleagues at the fitness center as well as teachers or personal trainers to obtain information about the kind of food to include or exclude from the diet, and the kind of ergogenic they should consume to get faster results (including anabolic steroids) [2,3,17].

## Discussion

This analysis was conducted to examine the nutritional characteristics of physically active individuals and their eating habits. Studies were grouped according to the most frequent categories of analysis. We found a small number of female participants in our review. This finding is consistent with other reports in the literature, which show a higher prevalence of men, both in physical activity with body burden for muscle hypertrophy and the consumption of steroids. Findings from this review highlight the concepts that the physically active individuals have about nutrition, which ultimately influence their eating habits in search of lean mass.

The main feeding characteristics of a person with muscle dysmorphia is the hyperprotein and hipolipidic diets with the consumption of dietary supplements in order to increase muscle mass and reduce body fat mass.

Pereira, Souza and Lisboa [13] investigated the dietary profile of bodybuilders at maturity and found that the most consumed foods among the carbohydrates were: pasta (88.9%), bread (88.9%), white rice (77.8%), oats (77.8%), sugar (55.5%), corn flakes (25%) and rice (22.2%). The protein sources were milk (88.8%), eggs (88.8%), beef (100%) and cheese (100%), fruit consumption per meal was 100% at breakfast, 22% in the collation, 12% at lunch and 22% in the snack, without consumption at dinner, while the consumption of vegetables was 89% only at lunch. The most consumed fruits were: papaya, banana, apple, orange, melon, pineapple, watermelon, plum, mango and tomato. The leafy vegetables were carrots, chayote, lettuce, green beans, chard, cabbage, and kale sheets. Azevedo et al. [3] concluded that the growing phenomenon of excessive and uncontrolled consumption of protein and the drastic reduction in lipid intake as a result of the excessive desire for aesthetic modification reflects a serious public health problem.

Behar and Molinari [2] tried to characterize the behavior of bodybuilders who changed their eating behaviors as a consequence of muscle dysmorphia. The study was done with two populations: 8 men weightlifters without history of eating disorders and 84 medical students who also lifted weight but had eating disorders. The results showed a prevalence of 13.6% of MD among weightlifters compared to no MD among medical students, who were less interested in the physical aspects. The weightlifters with MD monitored the weight more often, 83.3% were more concerned about the feeding schedule, all subjects with MD were concerned about the distribution of nutrients and 84.2% consumed special foods or ergogenic supplements. Duties as study, work, meetings and other commitments of social life were pushed aside by weight lifters due to their diet, a priority for these people, 42% were using anabolic steroids to increase muscle and 17% induced vomiting to avoid overweight. Among the group with eating disorders this percentage was 12% [2].

When investigating the prevalence of the use of dietary supplements by bodybuilders, Linhares and Lima [6] concluded that although food supplementation is indicated when the body needs complementary feeding, this practice is becoming increasingly common among strength training physically actives subjects, without guidance and without a specialized professional in order to obtain a defined musculature in a short time. Sardinha, Oliveira and Araujo investigated the association between MD, excessive practice of physical exercises to increase muscle mass, and the use of ergogenic aids and hyperprotein diets by physically active subjects and weight lifters [9]. A hundred men aged 18–35 years old in six academies in the north of Rio de Janeiro were investigated. All answered a questionnaire consisting of 12 items with questions about food consumption and MD (**what did they find**????).

In this context, Pope et al. [4] concluded that this inadequate feeding practice by MD men affected their resulted in making them were prone to suicide attempts; the use of dietary supplements and anabolic steroid abuse was linked to a higher frequency of disorders; and the lack of adequate food was linked to worsening of psychopathology and compromised. The authors reported that 14 men were identified with eating disorders like bulimia and anorexia nervosa [4].

Kanayama [11] investigated body image, attitudes toward male roles, eating habits and behaviors in physically active individuals who used steroid. The results showed that individuals who consumed steroids had their dietary habits modified so that the concepts about healthy eating based on food consumption as part of a balanced diet were distorted, because foods and micronutrients were being replaced by ergogenic supplements. The main reason for this replacement was the obsession with losing fat and gaining more muscles in less time, strengthening male roles and getting the perfect body portrayed by the media, resulting in less focus on optimal health by feeding [11].

16% of individuals studied by Sardinha et al. reported use of anabolic steroids and 45% reported using dietary supplements; 8% of the individuals were concomitant users of both types of substances. The survey found that most did not have a prescription and were not following any guidance by a nutritionist. However, the use of ergogenic aids by physically active individuals may be even higher, given that many stopped to declare such food practices spontaneously aiming MD [9].

Changes in the diet of bodybuilders, including dietary supplements used as ergogenic anabolic steroids for increasing skeletal muscle mass was investigated by Iriart, Keys and Orleans [1]. They found that both bodybuilders from middle class gyms and from the periphery were dissatisfied with their bodies and consumed anabolic steroids because they believed that the effects of a balanced diet for fat loss and muscle mass gain by exercise were very slow. In this sense, the general thinking was that anabolic steroids provided muscle mass faster gain [1].

Trog and Teixeira [7] found that many respondents, in addition to eating a smaller number of daily meals, sought to replace a balanced diet, rich in vegetables and fiber, and adequate amounts of carbohydrates, fats, and proteins with food supplements. The main reasons recorded in the survey were: to increase muscle mass (32%), to obtain rapid results (20%), more results (16%), to improve fitness (12%), to improve stamina and strength (12%) and to improve training (8%).

Proteins and amino acids were consumed by 84% of respondents, vitamins and minerals by 4%, and others by 4%. In this survey, 48% received professional guidance to consume the supplements, 28% used them by influence of friends, 8% under the influence of advertisements and 16% influenced by other reasons, such as internet searches. The study concluded that there is a need for recruitment and contract of permanent nutrition professionals by the academies as part of the services provided, focusing on highlighting the importance of food and nutrition issues for physically active subjects [7].

The most common anabolic products consumed by respondents were: Sustanon (proprionate/fenilproprionato/isocaproato/testosterone caproate), Deca-durabolim (decanoanato of landrolona) and Winstrol (stanozolol). There were also cases of use of Deposteron (testosterone cypionate), Primobolan (methenolone); Hemogenin (Anadrol) and veterinary products such as Androgenol (testosterone animal) and ADE (multivitamin). Increased use of veterinary products was found among users of popular academies, probably because of the ease of purchase, either for access or price, while the high income users have access and affordability to buy imported products [1,17].

The risks of indiscriminate consumption of food supplements, alone or in combination with other substances that may stimulate the central nervous system include: increased blood pressure and heart rate, propensity to cardiac arrhythmias, coronary spasm, and myocardial ischemia in susceptible people. Other symptoms include sleep disturbances, tremor, agitation, lack of coordination and psychological dependence [6].

The main finding of this study is that individuals engaged in physical activity who have muscle dysmorphia do not usually consult appropriate professionals to plan their diet. This feeding behavior is a risky behavior to the health and quality of life of these people. Thus, we plan interventions to identify these individuals and to change this situation. Understanding MD includes questioning regarding values related to body, beauty, attributes that dictate the beautiful, but also the culture related to the body in different political, economic and social contexts to understand, even from the moment that a lean and athletic body has become a standard of beauty. The combination of an unbalanced nutritional diet, based on the belief that practicing physical activities and the use of supplementary food, especially amino acids, and the tendency for the development of MD, causes image distortion by the individual's own body, resulting in ignoring the real image and pursuing an idealized image [3] due to the important role of the muscle system [18-21].

## Concluding remarks

Only by following the appropriate mix of macro and micronutrients in the diet according to the caloric expenditure and the exercises is that one can get lean. The role of the nutritionist is to guide the portion size, estimate the energy value of the diet and its distribution in macronutrient and consider the principles of nutrition to functional recovery of the digestive process, promote liver detoxification, balance and guide to organic adequate intake of supplemental nutrients and other substances. Thus, we strongly suggest an integrated work supported by professionals to reverse the MD condition.

## Competing interests

The authors declare that they have no competing interests.

## Authors’ contributions

All authors participated in the revision of the manuscript. NC, FA, MTB, CBMM, LCA, VEV, FSA, APL, MAC, JB, FAGV, CL and DESF determined the design, interpreted the text and drafted the manuscript. All authors read and gave final approval for the version submitted for publication.
